# Case report: A rare appearance of preretinal deposits in a patient with uveitis: multimodal imaging observation

**DOI:** 10.3389/fmed.2023.1121419

**Published:** 2023-08-08

**Authors:** Yizhe Cheng, Chunli Chen, Yuanyuan Xiao, Shuang Wang, Sihui Wang, Xiaoyan Peng

**Affiliations:** ^1^Department of Ophthalmology, Beijing Tongren Hospital, Capital Medical University, Beijing, China; ^2^Beijing Ophthalmology and Visual Science Key Laboratory, Beijing, China; ^3^Beijing Institute of Ophthalmology, Beijing, China; ^4^Department of Neuroradiology, Beijing Tiantan Hospital, Capital Medical University, Beijing, China

**Keywords:** uveitis, preretinal deposits, multimodal imaging, tubercular infection, dexamethasone intravitreal implant

## Abstract

**Background:**

Uveitis is a disease presenting with varied clinical symptoms and potentially devastates visual function. Here, we report a patient with uveitis exhibiting a rare appearance of preretinal deposits (PDs).

**Case presentation:**

A 49-year-old female showed vitreous opacity and perivascular white PDs involving veins and arteries. The interferon-gamma release assay was strongly positive and chest computed tomography showed signs of calcified nodules; other tests were unremarkable. The patient was diagnosed with uveitis and tubercular infection. The patient was given systemic anti-tubercular therapy and steroids, which were subsequently combined with immunosuppressants. The shrinkage of HRD was more sensitively observed with OCT than on photographs during follow-up visits. The right eye was relieved subsequently, but the left eye showed vitreous opacity and responded poorly to the treatment. Three months after the dexamethasone intravitreal implant, the perivascular deposits in the left eye disappeared and the vitreous opacity was relieved.

**Conclusion:**

PDs can appear as spotted deposits in the posterior pole and segmental deposits in the periphery in patients with uveitis, which mainly involves the vitreous cavity and is easily confused with retinal vasculitis. OCT can more sensitively observe the response than other examinations.

## Introduction

Uveitis is a common vision-threatening inflammatory ocular disease and includes multiple heterogeneous clinical entities (1) that form a collection of more than 30 diseases characterized by inflammation inside the eye (2). It accounts for 5–10% of visual impairments worldwide (1). Although some uveitis cases are linked to a systemic infection or a rheumatologic disease, many cases are presumed to be immune-mediated and without any known systemic associations (2, 3). Up until now, at least 50% of patients with uveitis had failed to find a specific etiology (1, 3).

Preretinal deposits (PDs), as far as we know, appear relatively rarely in uveitis. PDs were first reported by Nakao et al. in a cohort with human T cell lymphotropic virus type 1 (HTLV-I)-associated uveitis (HAU) in 1996. In Nakao’s study, eight of 55 cases exhibited gray-white granular PDs scattered on the retinal veins and/or arteries in the posterior pole. These PDs resolved in a few weeks spontaneously or in response to corticosteroids together with anterior uveal inflammation (4). Subsequently, PDs were increasingly reported in different diseases, including infectious diseases [ocular toxoplasmosis (OT) (5–11), syphilitic uveitis (12–16), vitreoretinal lymphoma (VRL) (8, 17–20), HAU or HTLV-I carriers (4, 21) and acute retinal necrosis (ARN) (22, 23), and endogenous fungal endophthalmites] (24–28), and uveitis without a certain etiology (9, 17). In some iatrogenic conditions, exogenous materials comprising silicone oil (SO) tamponade (29–33), intravitreal antibiotics (34, 35), and the suspicious vitreous remnant of a viscoelastic substance (36) can also present with PDs. However, few published articles mention and describe the interesting manifestation and there is insufficient imaging data exhibiting the features of PDs.

We report this case, which presented as uveitis, mainly affecting the vitreous cavity, with a rare appearance of PDs of inflammatory cells on the internal limiting membrane/epiretinal membrane (ILM/ERM). The deposits in the right eye were relieved after the 6-month oral steroid treatment along with anti-TB therapy (ATT). As far as we know, this manifestation of PDs has not been reported previously by multimodal imaging. The case was documented by multimodal imaging in a 25-month follow-up, continuously displaying the response to the treatment. In addition, the patient’s treatment may provide some references for similar cases.

## Case presentation

A 49-year-old female patient was referred to our clinic, complaining of a bilateral floating black shadow for approximately 4 months. There was no history of fever, backache, weight loss, hearing loss, mouth ulcers, skin lesions, or any other associated systemic illness. The patient’s sister-in-law had tuberculosis 10 years previously. The patient had not received a Bacillus Calmette-Guerin vaccine and had been diagnosed with capillary occlusion in another ophthalmology clinic.

**Figure 1:**
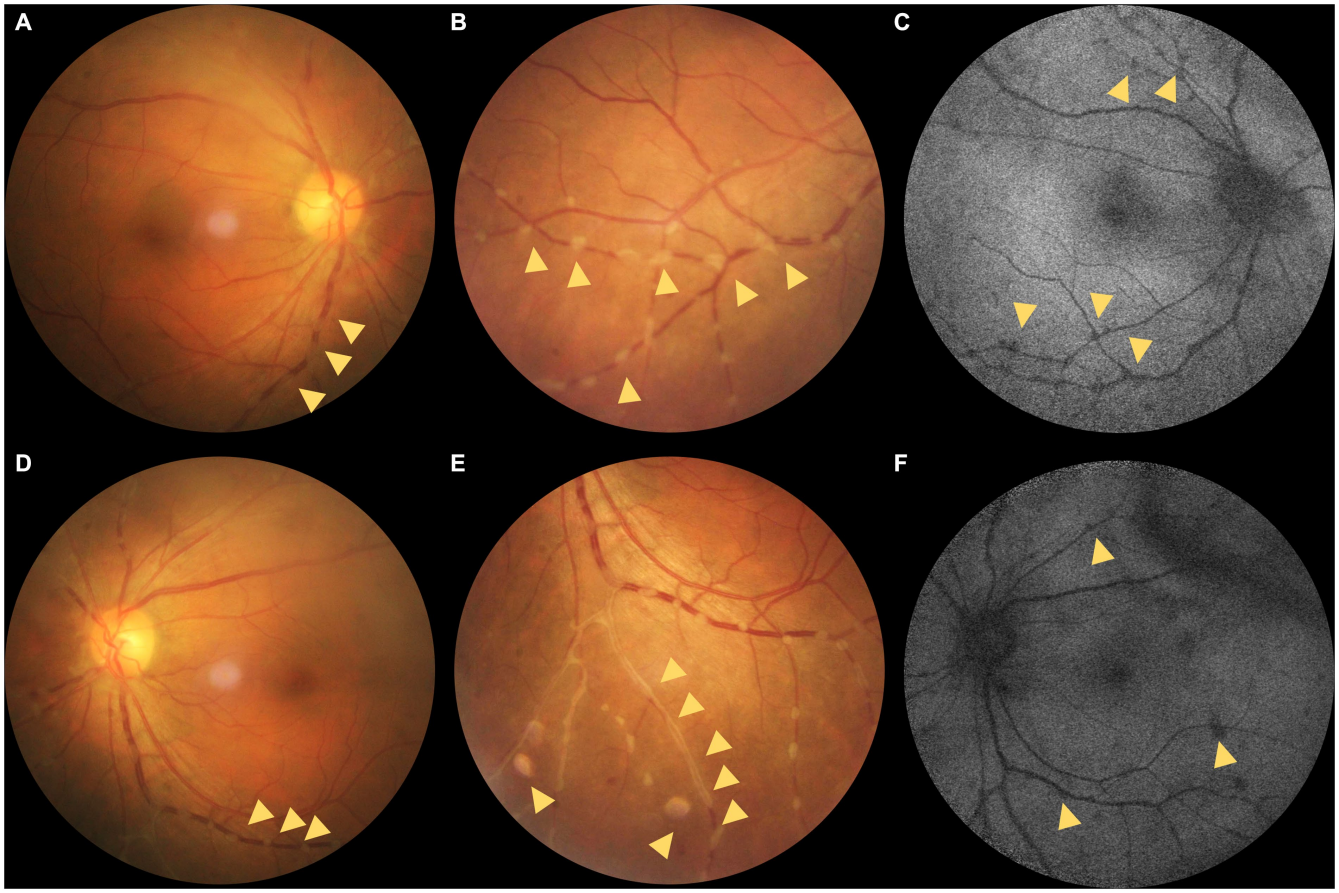
**(A–C)** Color fundus photographs (CFPs) and fundus autofluorescence (FAF) of the right eye. **(D–F)** CFPs and FAF of the left eye. **(A,B)** Dotted grayish-white deposits around the retinal vessels (yellow triangles; magnified in **B**). **(C)** Hypo-AF corresponding to the grayish-white deposits on the CFPs and mottled hypo-AF (yellow triangles). **(D,E)** Dotted grayish-white deposits around the retinal vessels. **(E)** Magnification of the dotted grayish-white deposits around the retinal vessels and segmental deposits around the peripheral vessels (yellow triangles). **(F)** Hypo-AF corresponding to the grayish-white deposits on the CFPs and linear hypo-AF consistent with the vitreous opacity (yellow triangles).

**Figure 2:**
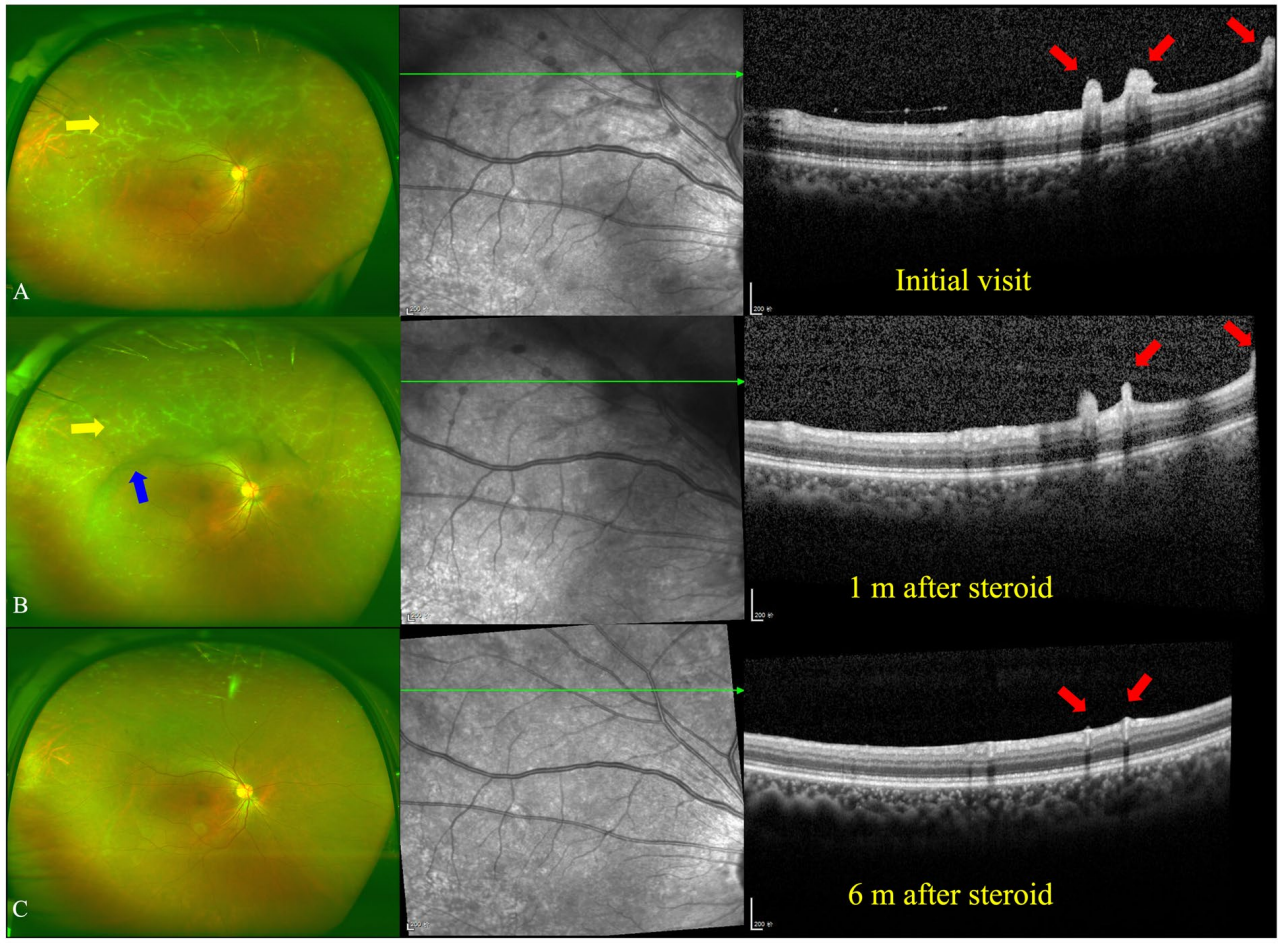
Ultra-wide fundus photograph (UWFP) and optical coherence tomography (OCT) of the right eye during follow up. **(A)** White deposits around the peripheral vessels and vitreous opacity on a UWFP and hump-like hyperreflective materials (HRMs) on the inner limiting membrane/epiretinal retinal membrane (ILM/ERM) at the initial visit. **(B)** White deposits around the peripheral vessels and vitreous opacity on a UWFP and shrinkage of hump-like HRMs on the ILM/ERM 1 month after oral steroid administration. **(C)** The disappearance of white deposits around the peripheral vessels and vitreous opacity on a UWFP and the disappearance of hump-like HRMs on the ILM/ERM 6 months after oral steroid administration. The yellow and blue arrows, respectively, indicate PDs and vitreous floaters on the UWFP, and the red arrows indicate PDs on the OCT.

**Figure 3:**
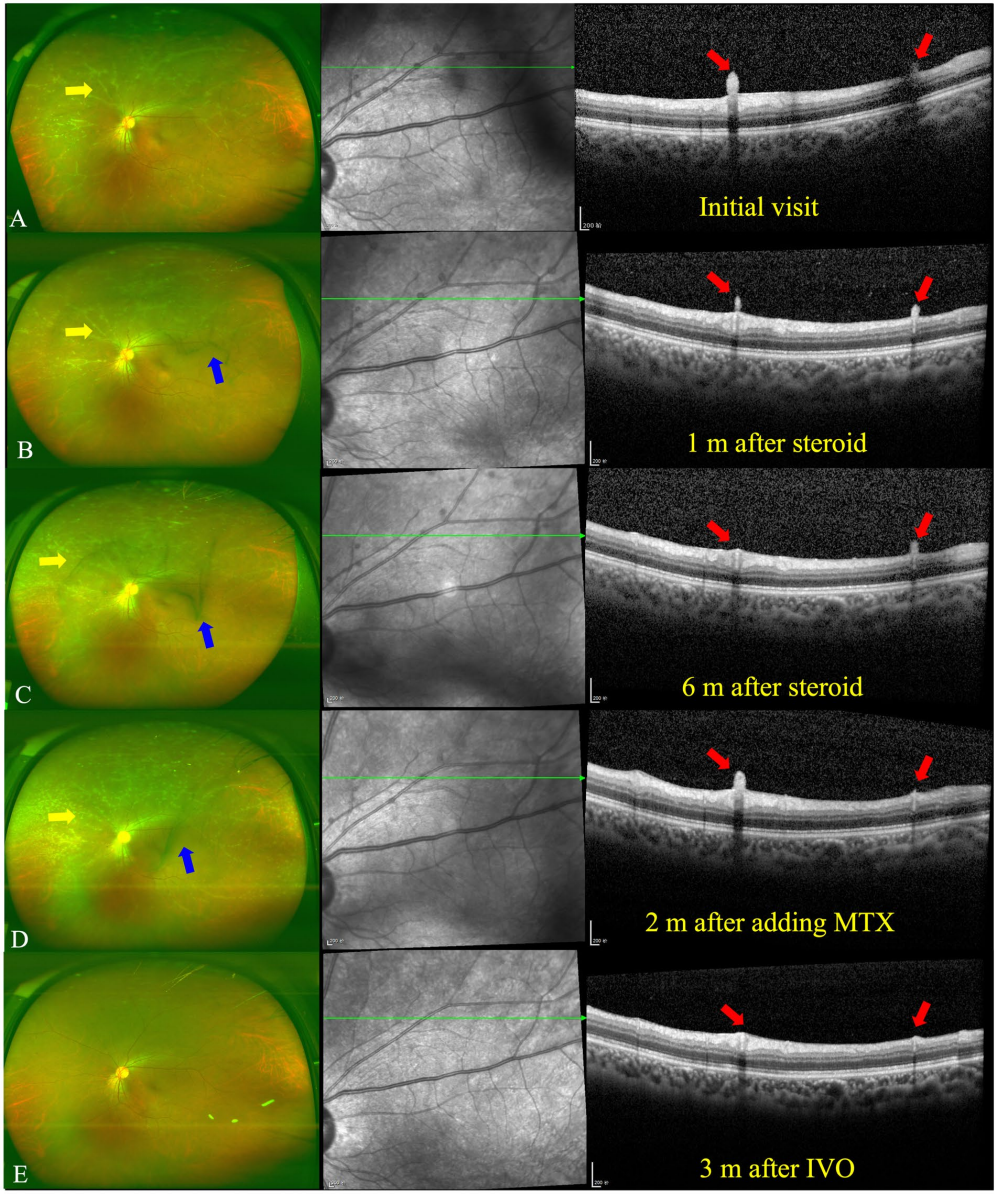
UWFP and OCT of the left eye at follow up. **(A)** White deposits around the peripheral vessels and vitreous opacity on a UWFP and hump-like HRMs on the ILM/ERM at the initial visit. **(B)** White deposits around the peripheral vessels and vitreous opacity on a UWFP and shrinkage of hump-like HRMs on the ILM/ERM 1 month after oral steroid administration. **(C)** White deposits around the peripheral vessels and vitreous opacity on a UWFP and further shrinkage of hump-like HRMs on the ILM/ERM 6 months after oral steroid administration. **(D)** Increased numbers of white deposits around the peripheral vessels and vitreous opacity on a UWFP and relapse of hump-like HRMs on the ILM/ERM 2 months after the addition of methotrexate (MTX). **(E)** The disappearance of white deposits around the peripheral vessels and vitreous opacity on a UWFP and the disappearance of hump-like HRMs on the ILM/ERM 3 months after a dexamethasone intravitreal implant (IVDI). The yellow and blue arrows, respectively, indicate PDs and vitreous floaters on the UWFP, and the red arrows indicate PDs on the OCT.

**Figure 4:**
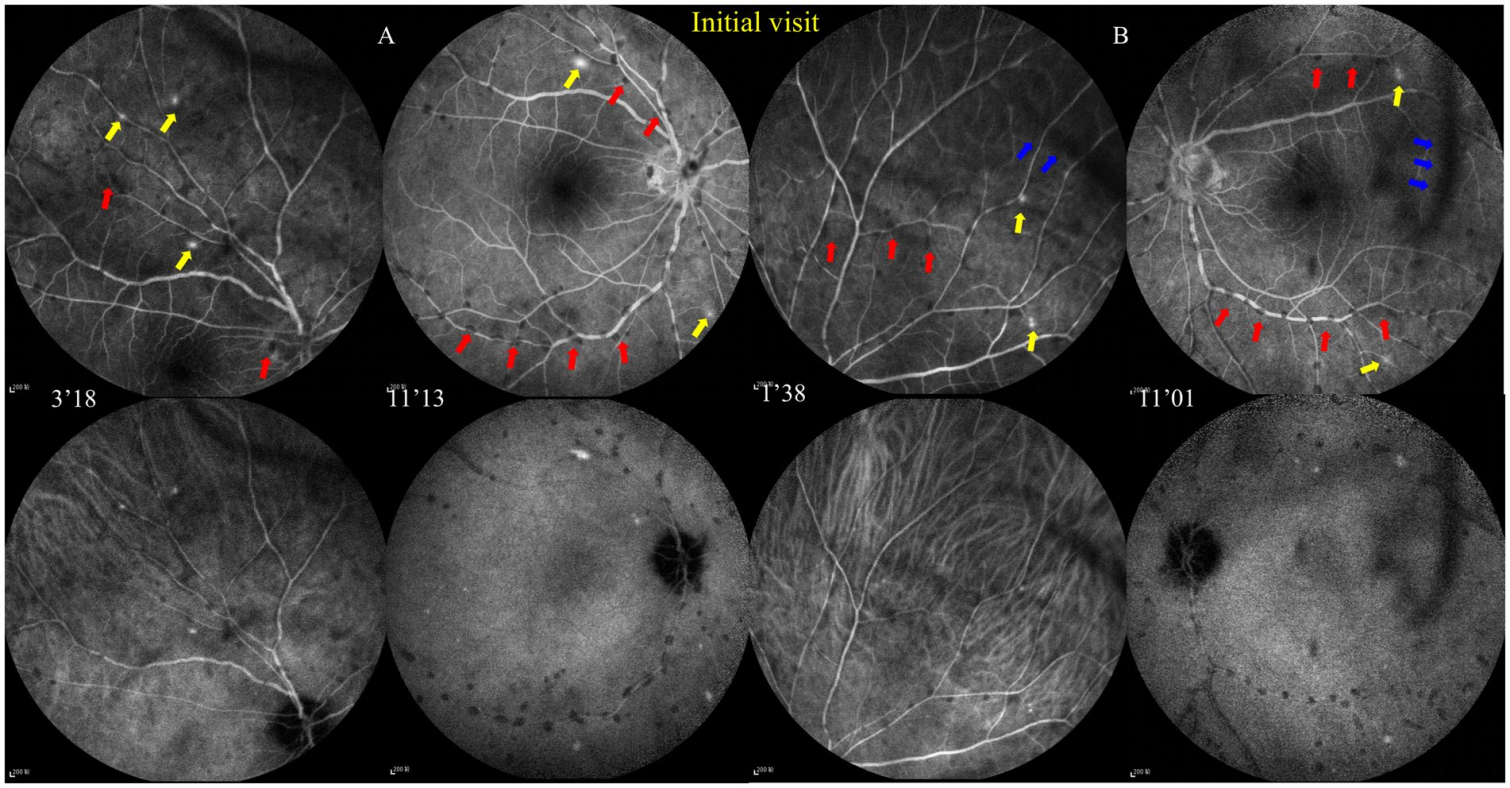
Fundus fluorescence angiography (FFA) and indocyanine green angiography (ICGA) at the initial visit. **(A,B)** FFA shows dotted perivascular hypofluorescence in the posterior pole and midperiphery and some hyperfluorescent dots in the midperiphery, which had leakage in the late phase, and hyperfluorescence of the optic disc was noted in both eyes with late FFA.ICGA shows that those hypofluorescent dots of the FFA remained hypofluorescent with ICGA. The red and blue arrows, respectively, indicate hypofluorescent PDs and vitreous floaters, and the yellow arrows indicate hyperfluorescent leakage.

The best corrected visual acuity (VA) was 20/20 in both eyes. Anterior segment examination was within normal limits in both eyes. The intraocular pressure (IOP) was 11.7 mmHg in the right eye and 11.6 mmHg in the left eye. A color fundus photograph (CFP) and ultra-wide fundus photograph (UWFP) of both eyes showed dotted and segmental perivascular grayish-white deposits involving major vessels in the posterior pole and midperiphery with vitreous opacity (Figures 1A,B,D,E, 2A, 3A). Fundus autofluorescence (FAF) showed dotted and segmental hypoautofluorescence around vessels corresponding to the deposits on the CFP (Figures 1C,F). Optical coherence tomography (OCT) showed hump-like elevated hyperreflective deposits (HRDs) in the interface of the retinal vessels and vitreous cavity (VC), and several clusters of hyperreflective materials (HRMs) in the VC (Figures 2A, 3A). Fundus fluorescence angiography (FFA) showed dotted perivascular hypofluorescence in the posterior pole and midperiphery throughout all the phases in both eyes. In addition, early FFA of both eyes showed some hyperfluorescent dots in the midperiphery, which leaked in the late phase. Hyperfluorescence of the optic disc was also noted in both eyes on late FFA (Figures 4A–D). Indocyanine green angiography (ICGA) served to exhibit clear shapes and the sites of these deposits, which had a hypofluorescent appearance (Figures 4E–H). The imaging findings of PDs are summarized in Supplementary Table S1.

The patient was investigated through a complete work-up, including complete blood count, a renal and liver function test, infectious diseases tests (syphilis, hepatitis B and C, and human immunodeficiency virus), serum angiotensin-converting enzyme, *Mycobacterium tuberculosis*-IFN-γ release assay (TB-IGRA), erythrocyte sedimentation rate, rheumatoid factor, anti-neutrophil cytoplasmic antibodies, and antinuclear antibody, which were within normal limits except for a positive TB-IGRA of 301.9 pg./mL (normally not more than 14 pg./mL). Toxoplasmosis IgG and IgM were within normal limits. The viral analysis of the aqueous humor, including cytomegalovirus (CMV), varicella-zoster virus (VZV), herpes simplex virus (HSV), and Epstein–Barr virus (EBV), revealed no positive results. Serum antibody of human T cell lymphotropic virus type 1 (HTLV-I) was supplementally conducted with no positive result. Purified protein derivative of tuberculin was performed in the local clinic and revealed that the local redness and induration was 15 mm in diameter and without necrosis and blisters. Computed tomography (CT) of the thorax showed well-defined nodules in the right upper lobe and the right middle lobe and nodular calcified lesions with lengths of approximately 5.5 mm in the posterior segment of the right upper lobe and nodular calcified lesions in the right hilum of the lung (Supplementary Figure S1) (the laboratory results are shown in Supplementary Table S2).

After consultation in the respiratory department, owing to TB infection, systemic anti-tubercular treatment was initiated with 600 mg of isoniazid, 300 mg of rifampicin, 1,500 mg of pyrazinamide, and 250 mg of levofloxacin along with oral prednisolone at 50 mg a day (the body weight of the patient was 76.8 kg). At the 1-month follow-up, a fundus examination of both eyes showed that the floaters in the VC were still present. However, the shrinkage of the HRDs was noted by OCT in both eyes (Figures 2B, 3B).

At the 6-month follow-up, the steroid was gradually tapered and remained at 7.5 mg. Fundus examination of the right eye showed both perivascular grayish-white deposits and the black floaters in the VC had disappeared. In addition, the HRDs of the right eye resolved and no hyperreflective materials were found in the VC with OCT (Figure 2C). However, in the left eye, the grayish-white deposits seemed to occur much more in the nasal periphery on the UWFP and the size of the HRDs became smaller than before and even disappeared at some sites. The black floats in the VC were much larger than before (Figure 3C). On FFA, the fluorescence leakage and staining of vessels could be noted in the periphery of both eyes. Additionally, a hot optic disc was observed in the late phase for both eyes. Furthermore, the hypofluorescence of PDs and vitreous opacity could be found in the left eye (Supplementary Figure S2).

At the 10-month follow-up, the patient complained of a decreased VA in the left eye. The systemic anti-tubercular treatment was accordingly changed to a dual anti-tubercular plan with the guidance of the respiratory department. Owing to the long-term use of systematic steroids and unilaterally decreased VA, 0.5 mL of 40 mg/mL triamcinolone acetonide (TA) sterile suspension (Kenacort-A, Jida Pharmac, Kunming, China) mixed with 0.5 mL of 2% lidocaine was slowly injected 1.5 cm into the orbit along the orbital margin of the lower eyelid. TA was administered in the presence of no blood in the pumpback after insertion to the left eye.

At the 11-month follow-up, the patient said that the symptoms in the left eye were relieved with the improvement of VA, suggesting the effectiveness of regional steroids. However, the regional steroids of TA might just work for approximately 2 to 3 weeks. So, methotrexate (MTX) was initiated as a steroid-sparing therapy in a weekly dose of 7.5 mg after an evaluation of liver and renal function. Another retrobulbar injection of TA was administered for supplementary anti-inflammatory therapy because MTX takes 6 to 8 weeks to have an effect. We enjoined the patient’s steroid tapering to 5 mg per day beyond 6 weeks after adding MTX.

At the 13-month follow-up, the right eye remained stationary when the patient took MTX for 2 months. By contrast, the grayish-white deposits of the left eye increased and the vitreous floaters were not improved (Figure 3D). MTX was increased to 10 mg per week. Then, 0.7 mg of dexamethasone intravitreal implant (Ozurdex, Allergan, Irvine, CA, United States) was injected for the left eye, given the previously effective response of regional steroids. In addition, the patient showed normal intraocular pressure (IOP) after regional steroids and a dexamethasone implant. Notably, 3 months after the intravitreal dexamethasone implant (IVDI) for the left eye, the deposits disappeared on the UWFP, and the HRDs were resolved correspondingly (Figure 3E).

At the 20-month follow-up, fundus examinations of both eyes showed restoration and a quiet condition. FFA and ICGA were performed again. The early FFA showed mottled hyperfluorescence in the posterior pole and periphery. The late FFA revealed mild leakage of the deep capillaries. The late ICGA showed hyperfluorescence corresponding to the hyperfluorescence on the FFA (Supplementary Figure S3). Owing to the mild leakage of the left eye, we recommended another IVDI for the patient.

At the 25-month follow-up, FFA showed the leakage level of the left eye had been relieved. There was only capillary leakage in the posterior pole and midperiphery (Supplementary Figure S4). MTX was maintained for 14 months with regular liver and renal function monitoring. OCT showed no macular abnormalities, except for the epiretinal membrane. The patient showed no secondary complications and there was a clear improvement in the previous symptoms at the last follow-up. The changes in VA and IOP are depicted as line graphs in Supplementary Figure S5.

## Discussion

The diagnosis of our patient was uveitis and tubercular infection. Although several studies have made the diagnosis of tubercular uveitis (TBU) based on TB-IGRA and/or a tuberculin skin test and/or polymerase chain reaction test in the presence of uveitis, there is no direct evidence (such as TB culture or TB-DNA positive) to approve the diagnosis of TBU in our patient, and the prevalence of TB infection remained at a relatively high proportion in the healthy population (37–39). The combination of a positive TB-IGRA, chest CT (showing nodular calcified lesions in the posterior segment of the right upper lobe and in the right hilum of the lung, where TB is preferentially involved), and a history of contact with a TB patient probably caused the previous pulmonary TB in our patient. In addition, the work-up for the exclusion of other cases of uveitis with certain etiologies showed no positive findings. Although the polymerase chain reaction test of TB-DNA was not conducted for this patient due to the low positive rate in our country and invasiveness of the sample-acquiring operation, ocular immunologic reaction induced by TB infection is a possible explanation; other etiologies that we cannot confirm are also probable.

The PDs in our patients are grayish-white, and diffusely scattered dots adhere to the retinal veins and arteries. In our review of the literature, we found that this perivascular pattern could be visualized in patients with OT, HAU, or ARN. The PDs of OT patients show multiple patterns, including an oval appearance, an isolated foveal residence, and a diffuse perivascular pattern, whereas HTLV-I carriers or ARN patients were reported to have a specific perivascular pattern. Nakao et al. initially documented these perivascular changes in HAU patients and HTLV-I carriers (4, 21). Mya Thida Ohn et al. reported three cases with ARN induced by the herpes zoster virus, exhibiting perivascular PDs, documented by FFA and multiple color photographs, that were very similar to those of our patients (23). However, the serum HTLV-I test was negative for our patient, and we analyzed their aqueous humor. No viral DNA debris was detected. Furthermore, we found that the PDs in patients with infectious conditions, such as toxoplasmosis and syphilis, are often accompanied by areas of retinitis, but there was no retinitis in any of the available imaging of our patient and no infection was found by laboratory tests. Govindahari et al. reported a case of preretinal exudates in ocular tuberculosis (40). Although the same causes of tubercular immunologic reactions were possible, the appearance of PDs and the types of uveitis in our case are different from Govindahari’s case. The PDs in Govindahari’s case were irregular (sheet-like or dot-like) and located randomly. The PDs in our case are adhered with the vascular and mostly in dotted appearance. Govindahari’s case exhibited mid-peripheral periphlebitis and healed chorioretinitis lesions. Our case only showed mild microvascular leakage of microvascular with FFA. More importantly, our case showed our experience in dealing with PDs, which has a poor response to oral corticosteroids. On all accounts, it is difficult to differentiate between our patient and other uveitis cases only by the morphology of PDs. The imaging findings accompanied by PDs and laboratory tests are important for providing clues when dealing with PDs. For example, yellowish PDs in conjunction with an underlying area of ground-glass retinitis are highly suggestive of ocular syphilis. Retinal hyperreflective round PDs, in conjunction with sub-lesional choroidal thickening and sub-lesional retinal pigment epithelium elevation, are more likely to occur in OT patients. However, the morphology of the PDs themselves can also provide some direction to our diagnosis. When we found perivascular PDs in a patient, we should take OT, ARN, HAU, or TB into account. More importantly, the laboratory tests will verify our ideas. The biggest barrier to summarizing the PD features of different entities is the uncertainty of the nature of the PDs. Therefore, cellular component analysis of PDs will be meaningful for future studies.

The manifestation that we are most interested in is the perivascular white deposits on the inner limiting membrane (ILM). However, we do not know the property of these materials. To determine whether the materials are exudations or deposits, we carefully compare the different manifestations in the fundus image, OCT, and FFA. With fundus images, it is difficult to differentiate exudations from deposits, as the materials stick to the vessels, and we are not able to find the origin of the materials on planar graphs. The only thing we can learn from the images is that the materials are in front of the retina. With OCT, three clues hint at the property of the materials. First, at the initial visit and 1-month follow-up (Figures 2A,B, 3A,B), hyperreflective dots were observed in the vitreous cavity. When these dots gradually reduced, the hump-like hyperreflective materials (HRMs) subsequently shrunk. Second, owing to the presence of the epiretinal retinal membrane (ERM), a hyperreflective separated line could be found between the hump-like hyperreflective materials and retinal tissue. Third, the HRMs on the left side in Figure 3B seemed to pile up and not directly connect with the retinal tissue due to the less reflective signals of the other materials. With FFA, even though there is a degree of capillary leakage in the deep retina and hyperfluorescent dots on the late FFA and ICGA, there was no obvious leakage of the retinal major arteries and veins, and no obvious staining of vessels was observed. Therefore, we speculate that the materials are not mainly from the retinal arteries and veins but the VC. The PDs in our patients seemed to have low mobility; therefore, we could observe the PDs in the fixed location using OCT. Additionally, the appearance of the deposit was easily confused with retinal vasculitis. Nevertheless, in our patient, there was no staining and obvious leakage of retinal vessels, as well as no hemorrhage and non-perfusion areas on the FFA, which are commonly regarded as typical features of retinal vasculitis (41).

Of note, PDs have some similarities with Kyrieleis plaques, which were previously referred to as segmental retinal periarteritis. Kyrieleis plaques were first described by Kyrieleis in 1933 in a case with presumed TBU (42), which is characterized by focal or glistening yellow-white accumulations surrounding the retinal arteries, particularly near areas of active retinal infection or inflammation (43). Kyrieleis plaques share a similar disease spectrum as PDs, including TRC (44, 45), TBU, syphilitic uveitis (46), and VZV and CMV infections (47, 48). Both PDs and Kyrieleis plaques represent inflammatory signs, most of which can be fully recovered and do not worsen the prognosis. More importantly, they both have a preference for retinal vessels. However, PDs are still different from Kyrieleis plaques in the following ways (49). First, the imaging appearance of PDs is different from Kyrieleis plaques. On the ophthalmoscope, PDs are mildly transparent grayish-white or yellowish-white dots, while Kyrieleis plaques are glistening yellowish-white accumulations, with high reflectivity of the affected vessel walls. In addition, PDs can involve both retinal arteries and veins, whereas Kyrieleis plaques affect only the retinal arteries. On FAF, FFA, and ICGA, PDs show hypofluorescence throughout all the frames. By contrast, Kyrieleis plaques show increased autofluorescence on FAF and early hypofluorescence and late hyperfluorescence on FFA and ICGA (50). Second, PDs are located in the interface of the retina and VC, which is confirmed by OCT, whereas the inflammation of Kyrieleis plaques affect only within the vessel walls and do not show any involvement outside the vessel walls (50). Third, PDs can completely resolve after treatment, in contrast to Kyrieleis plaques, which persist clinically with perivascular sheathing in some patients despite the resolution of chorioretinitis after antibiotic treatment (50).

So far, the nature of the preretinal deposits in our case has not been determined. It is suggested that they are clots of inflammatory cells, representing a specific inflammatory response to different stimuli (6, 21, 23). Paulus et al. reported the pathologic and cytologic findings of preretinal condensations in two patients with chronic uveitis, revealing the only presence of inflammatory cells (17). Calles Monar et al. believed the white cells could migrate through the retinal vessels into the vitreous and proposed that the deposits described in the avascular fovea are similar to precipitates in the corneal endothelium (9). However, in our patient, the PDs seem not to be from the retinal vessels but the VC. In addition, Nakao et al. provided a theory that may apply to the perivascular pattern of PDs (21). Nakao et al. believed that the activated T lymphocytes adhere to and migrate from the endothelium of retinal vessels and proliferate spontaneously. In this patient, we have excluded all the probable causes that were reported in the previous literature. TB infection is an exclusive cause. A similar phenomenon has been observed in patients with HTLV-I-associated CNS disease (21). The inflammation in TB uveitis is largely induced by activated T lymphocytes, instead of direct TB infection. The activated T lymphocytes may be inclined to form a perivascular pattern. Although our patient has no evidence of HTLV-I infection, the T lymphocytes activated by *Mycobacterium tuberculosis* are persistent in the immune system as memory T lymphocytes. It is possible that the lymphocytes adhere more readily to the blood vessels than to the retina and so would have a tendency to cluster along the retinal vessels. Taken together, we hypothesize that these materials are the inflammatory cells, probably T lymphocytes accumulating in a lump shape on the ERM/ILM.

## Data availability statement

The original contributions presented in the study are included in the article/Supplementary material, further inquiries can be directed to the corresponding author.

## Ethics statement

The studies involving human participants were reviewed and approved by Ethical committee of Beijing Tongren Hospital. The patients/participants provided their written informed consent to participate in this study. Written informed consent was obtained from the individual (s) for the publication of any potentially identifiable images or data included in this article.

## Author contributions

YC: writing and draft preparation. CC and XP: revisions. YX, ShW, and SiW: conceptualization. XP: review and editing. All authors read and approved the manuscript. All authors contributed to the article and approved the submitted version.

## Funding

This study was supported by The Capital Health Research and Development of Special (no SF-2018-2-1081) and the National Natural Science Foundation of China (82171073). The funding organizations had no role in the design or conduct of this research.

## Conflict of interest

The authors declare that the research was conducted in the absence of any commercial or financial relationships that could be construed as a potential conflict of interest.

## Publisher’s note

All claims expressed in this article are solely those of the authors and do not necessarily represent those of their affiliated organizations, or those of the publisher, the editors and the reviewers. Any product that may be evaluated in this article, or claim that may be made by its manufacturer, is not guaranteed or endorsed by the publisher.
