# Supplementary material for: Case report: A rare appearance of preretinal deposits in a patient with uveitis: multimodal imaging observation
Source: Front Med (Lausanne). 2023 Aug 8;10:1121419. doi: 10.3389/fmed.2023.1121419 (PMC10442564; doi:10.3389/fmed.2023.1121419)
Supplement: Supplementary file 1 [file Data_Sheet_1.docx]

TABLE S1 Multimodal imaging findings of preretinal deposits in the patient.

| Imaging examination | Features |
| --- | --- |
| CFP | Dotted and segmental perivascular grayish-white deposits involving major vessels |
| FAF | Dotted and segmental hypoautofluorescence around vessels |
| OCT | Hump-like elevated hyperreflective deposits in the interface of retinal vessels and vitreous cavity |
| FFA | Perivascular hypofluorescence throughout all the phases |
| ICGA | Clear shapes and sites of these deposits in a hypofluorescent appearance |

CFP = color fundus photograph, FAF = fundus autofluorescence, OCT = optical coherence tomography, FFA = fundus fluorescence angiography, ICGA = indocyanine green angiography.

TABLE S2. Laboratory results of serological and intraocular fluids test.

| Serological tests | |  |  |
| --- | --- | --- | --- |
| Complete blood count | |  | Reference |
|  | White blood cell | 6.4*10^9^/L | 3.5-9.5 |
|  | Red blood cell | 4.9*10^12^/L | 3.8-5.1 |
|  | Hemoglobin150 | 150g/L | 115-150 |
|  | Blood platelet | 216*10^9^/L | 100-350 |
| Renal and liver function test | | |  |
|  | Urea | 5.71 mmol/L | 2.86-8.2 |
|  | Creatinine | 55.0 umol/L | 44-97 |
|  | Uric acid | 471 umol/L | 89-416 |
|  | ALT | 12 U/L | 0-45 |
|  | AST | 15 U/L | 0-40 |
|  | AST/ALT | 1.25 |  |
|  | GGT | 15 U/L | 0-55 |
|  | ALP | 44 U/L | 25-150 |
|  | LDH | 183 U/L | 80-285 |
|  | TBIL | 11.6 umol/L | 3.4-25.4 |
|  | DBIL | 3.6 umol/L | 0-6.84 |
|  | IBIL | 8.0 umol/L | 0-14 |
|  | TP | 75.1 g/L | 62-85 |
|  | Albumin | 45.3 g/L | 35-54 |
|  | Globulin | 29.8 g/L | 20-40 |
|  | Albumin/Globulin | 1.52 | 1-2.5 |
|  | ADA | 7.0 U/L | 0-25 |
|  | TBA | 4.0 umol/L | 0-10 |
|  | Cholinesterase | 8459 U/L | 4000-13000 |
| Infectious diseases tests | |  |  |
|  | HBsAg | (-) 0.00 IU/mL | <0.05 |
|  | Syphilis specific antibody | (-) 0.05 | <1 |
|  | HIV combin | (-) 0.15 | <1 |
|  | HCVAb | (-) 0.04 | <1 |
| ACE |  | 36 U/L | 10--55 |
| ESR |  | 2 mm/h | 0--20mm/h |
| CRP |  | 3 mg/L | 0--8 |
| CYSTATIN C | | 0.7 mg/L | 0.54--1.15 |
| RF |  | 4.7 IU/mL | 0--20 |
| Complement 3 | | 128.4 mg/dl | 90--180 |
| Complement 4 | | 31.6 mg/dl | 10--40 |
| Mycobacterium tuberculosis-IFN-γ release assay | | | |
|  | Base level | 9.5 pg/ml |  |
|  | Stimulating level | 311.4 pg/ml | |
|  | TB-IGRA | 301.9 pg/ml (+) | 0-14 |
| Antinuclear antibody | | - | <1:100 |
| Anti-neutrophil cytoplasmic antibodies | | | |
|  | anti-PR3 antibody | <20 ru/ml | 0--20 |
|  | anti-MPO antibody | <20 ru/ml | 0--20 |
| HLA-B27 |  | (-) | (-） |
| Torch-combin | |  |  |
|  | Tox-IgG | (-) | (-) |
|  | Tox-IgM | (-) | (-) |
|  | RV-IgG | 2.16 U/mL | <1 |
|  | RV-IgM | (-) | (-) |
|  | CMV-IgG | 22.39 U/mL | <1 |
|  | CMV-IgM | (-) | (-) |
|  | HSV-IgG | 13.72 U/mL | <1 |
|  | HSV Ⅱ-IgG | (-) | (-) |
| HTLV Ⅰ& Ⅱ antibody | | (-) | (-) |
| Intraocular fluids test | |  |  |
|  | Copies (PCR test) | |  |
| CMV | 0 |  |  |
| HSV | 0 |  |  |
| VZV | 0 |  |  |
| EBV | 0 |  |  |

ALT = glutathione aminotransferase, AST = glutathione aminotransferase, GGT = glutamyl transpeptidase, ALP = alkaline phosphatase, LDH = lactate dehydrogenase, TBIL = total bilirubin, DBIL = direct bilirubin, IBIL = indirect bilirubin, TP = total protein, ADA = adenosine deaminase, TBA = total bile acids, HBaAg = hepatitis B surface antigen, TPHA = treponema pallidum particle agglutination test, hepatitis C antibody, ACE = angiotensin-converting enzyme, ESR = erythrocyte sedimentation rate, CRP = C-reactive protein, RF = rheumatoid factor, TB-IGRA = tuberculosis-IFN-γ release assay, Tox = Toxoplasma, RV = Rubella virus, CMV = Cytomegalovirus, HSV = Herpes simplex virus, HTLV-Ⅰ= Human T cell lymphotropic virus type 1, VZV = Varicella zoster virus, EBV = Epstein-Barr virus.


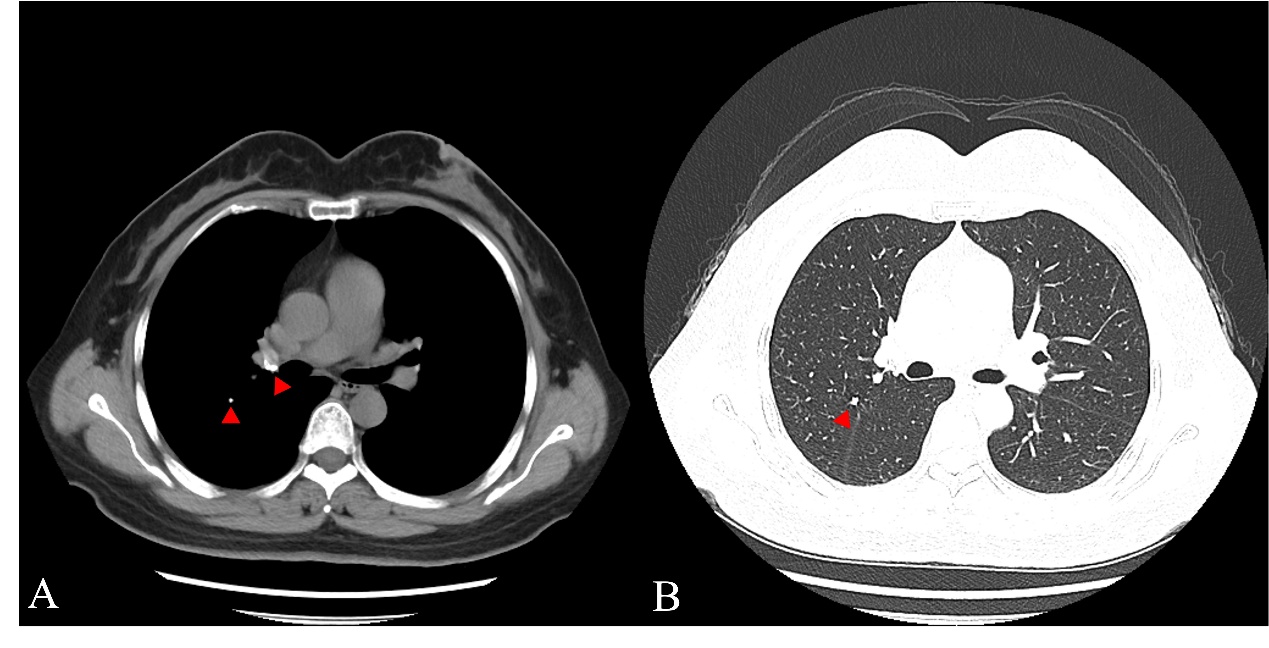


FIGURE S1. Thorax computed tomography images of the patient. **(A,B)** well-defined nodules in the right lobe (bottom red triangle in A and red triangle in B) and nodular calcified lesions in the right hilum of the lung (upper red triangle in A).


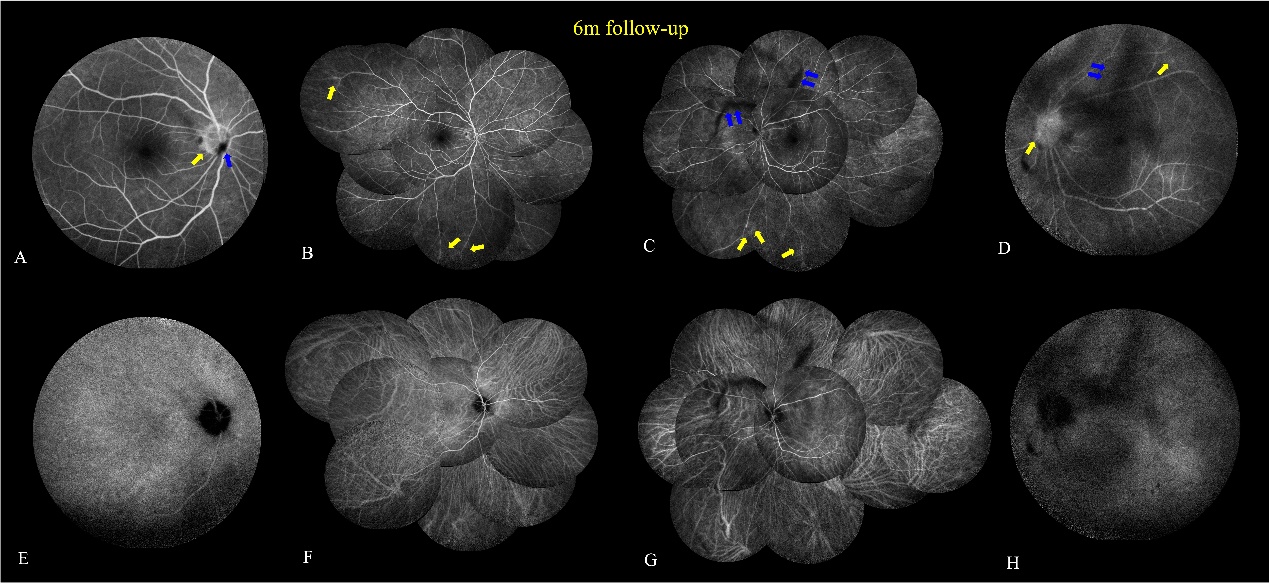


FIGURE S2. FFA and ICGA at the 6-month follow-up. **(A,B)** The FFA of the right eye showed hypofluorescent vitreous floaters and few sites of leakage. **(C,D)** The FFA of the left eye showed diffuse hypofluorescent vitreous floaters and multifocal leakage, and D showed a hot optic disc in the late frame. **(E,F)** The ICGA of the right eye showed no obvious abnormalities. **(G,H)** The ICGA of the left eye showed a hypofluorescent obstructing shadow of vitreous floaters. The blue arrows indicate hypofluorescent vitreous floaters, and the yellow arrows indicate hyperfluorescent leakage.


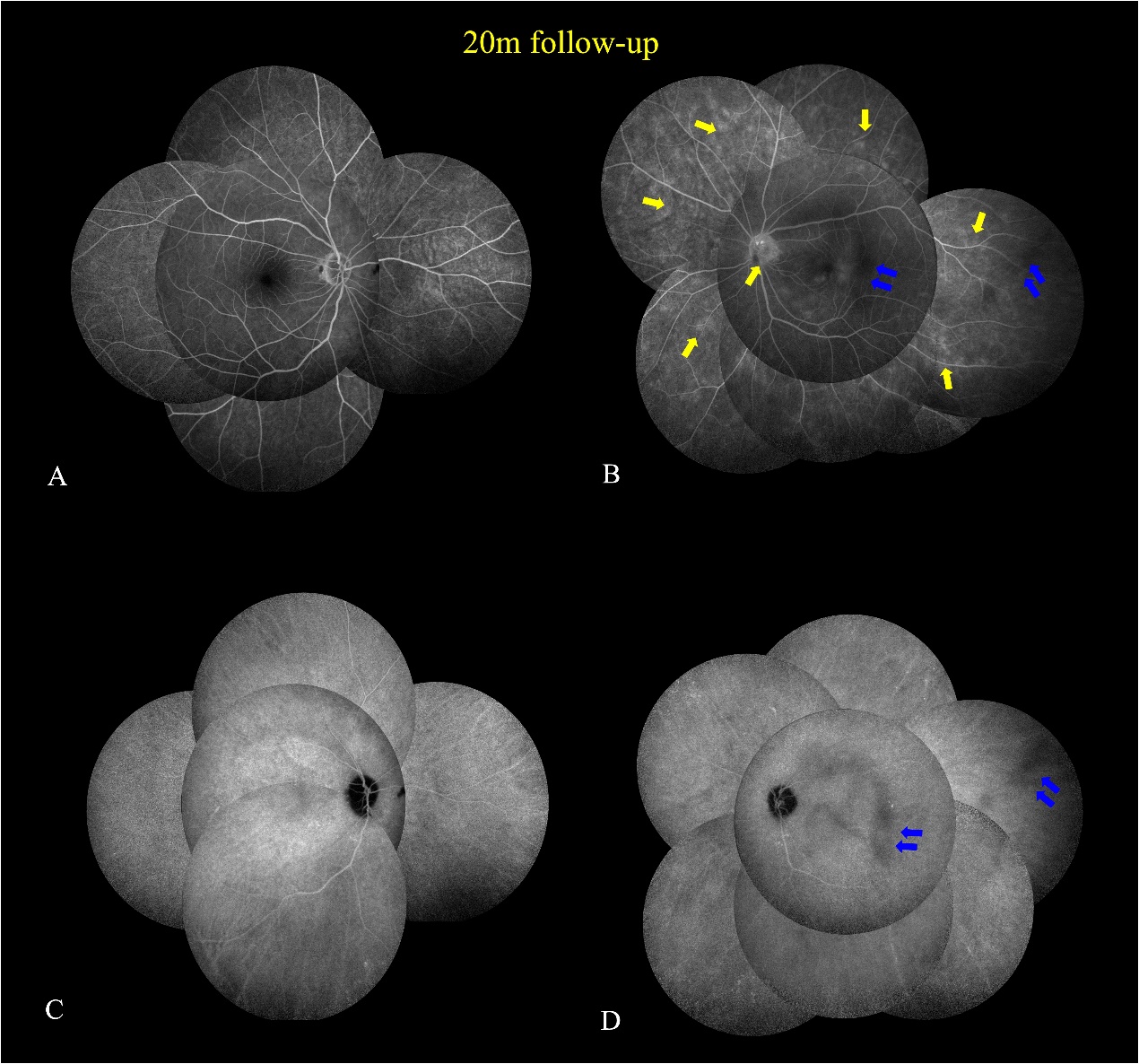


FIGURE S3. FFA and ICGA at the 20-month follow-up. **(A,B)** The FFA of the right eye showed no obvious abnormalities. The FFA of the left eye showed diffuse hypofluorescent vitreous floaters, multifocal leakage, and a hot optic disc in the late frame. **(C,D)** The ICGA of the right eye showed no obvious abnormalities. The ICGA of the left eye showed a hypofluorescent obstructing shadow of vitreous floaters. The blue arrows refer to hypofluorescent vitreous floaters, and the yellow arrows refer to hyperfluorescent leakage.


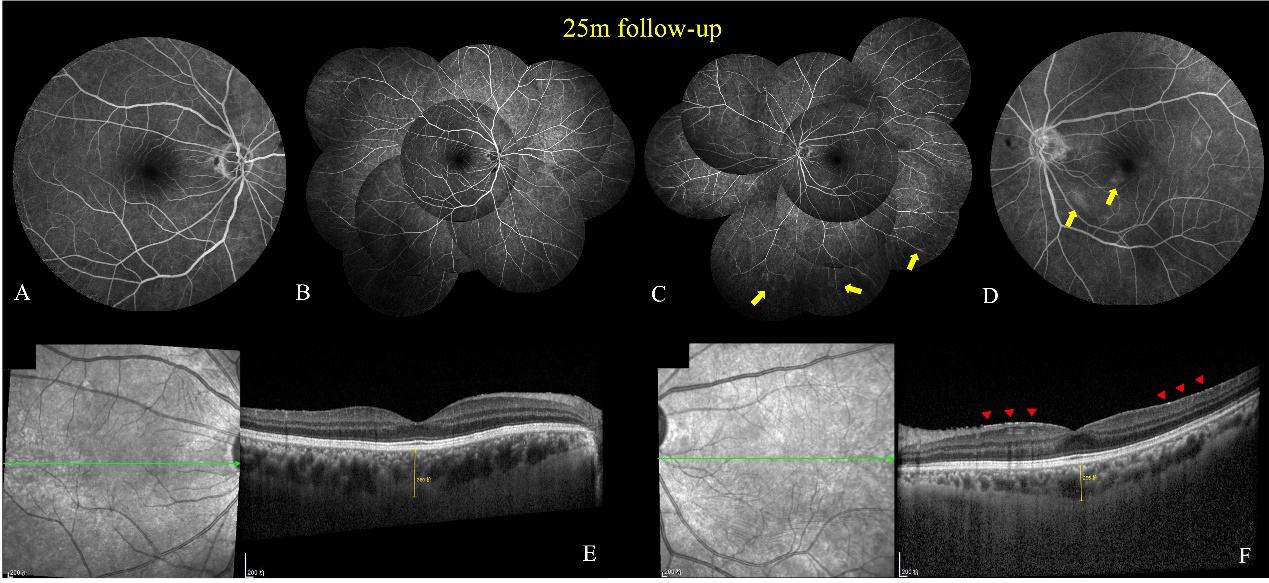


FIGURE S4. FFA and OCT at the 25-month follow-up visit. **(A,B)** The FFA of the right eye showed no obvious abnormalities. **(C,D)** The FFA of the left eye showed leakage in the posterior pole and midperiphery (yellow arrows). **(E,F)** OCT showed no obvious abnormalities in the right eye, but the presence of epiretinal membrane in the left eye (red triangle).


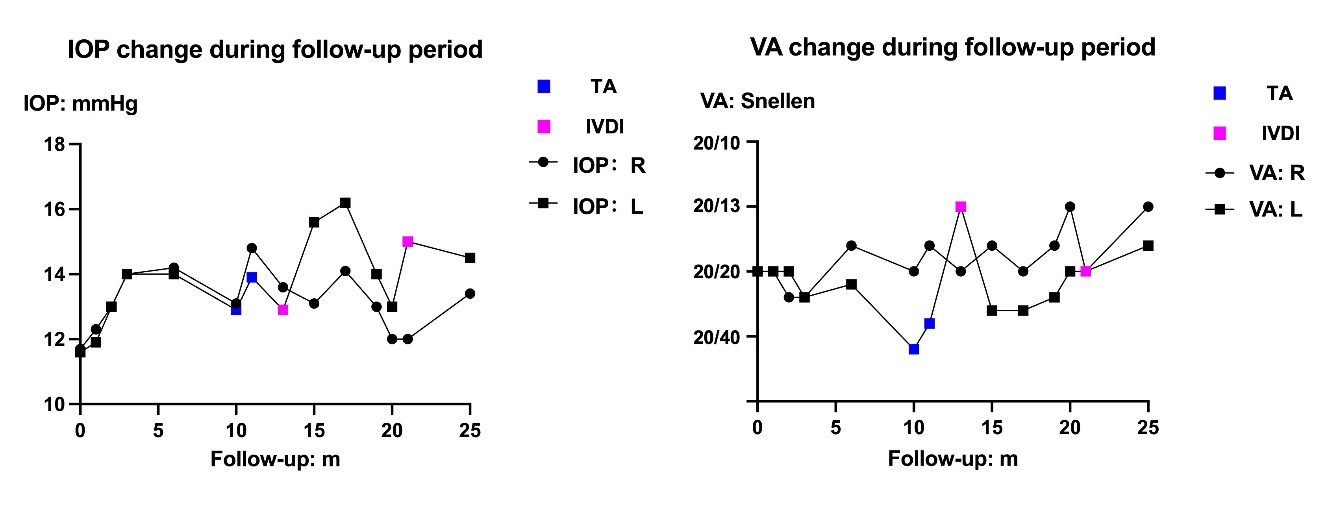


FIGURE S5. Changes in VA and IOP during the follow-up period. The magenta squares refer to the time when the patient received a TA injection, and the blue squares refer to the time when the patient received IVDI.
